# Changes in Lysozyme Flexibility upon Mutation Are Frequent, Large and Long-Ranged

**DOI:** 10.1371/journal.pcbi.1002409

**Published:** 2012-03-01

**Authors:** Deeptak Verma, Donald J. Jacobs, Dennis R. Livesay

**Affiliations:** 1 Department of Bioinformatics and Genomics, University of North Carolina at Charlotte, Charlotte, North Carolina, United States of America; 2 Department of Physics and Optical Science, University of North Carolina at Charlotte, Charlotte, North Carolina, United States of America; National Cancer Institute, United States of America and Tel Aviv University, Israel, United States of America

## Abstract

We investigate changes in human c-type lysozyme flexibility upon mutation via a Distance Constraint Model, which gives a statistical mechanical treatment of network rigidity. Specifically, two dynamical metrics are tracked. Changes in flexibility index quantify differences within backbone flexibility, whereas changes in the cooperativity correlation quantify differences within pairwise mechanical couplings. Regardless of metric, the same general conclusions are drawn. That is, small structural perturbations introduced by single point mutations have a frequent and pronounced affect on lysozyme flexibility that can extend over long distances. Specifically, an appreciable change occurs in backbone flexibility for 48% of the residues, and a change in cooperativity occurs in 42% of residue pairs. The average distance from mutation to a site with a change in flexibility is 17–20 Å. Interestingly, the frequency and scale of the changes within single point mutant structures are generally larger than those observed in the hen egg white lysozyme (HEWL) ortholog, which shares 61% sequence identity with human lysozyme. For example, point mutations often lead to substantial flexibility increases within the β-subdomain, which is consistent with experimental results indicating that it is the nucleation site for amyloid formation. However, β-subdomain flexibility within the human and HEWL orthologs is more similar despite the lowered sequence identity. These results suggest compensating mutations in HEWL reestablish desired properties.

## Author Summary

The functional importance of protein dynamics is universally accepted, making the study of dynamical similarities and differences among proteins of the same function an intriguing problem. While some metrics are likely to be conserved across family, differences are also very common. In previous works we have used a Distance Constraint Model to quantify flexibility differences across sets of orthologous proteins, which reproduce this diversity. In the same manner, this work investigates changes occurring upon individual point mutations. Somewhat surprisingly, the small structural perturbations caused by mutation lead to changes throughout the protein. These changes can be quite large, actually surpassing the scale for differences between ortholog pairs. Moreover, changes in flexibility frequently occur at sites far from the mutation site. These results underscore the sensitivity of protein dynamics in connection with allostery, and help explain why differences across protein families are so common.

## Introduction

Protein dynamics are intimately related to functional mechanisms [1], and changes therein can lead to observable phenotypes and disease [2]. These changes can be subtle. For example, a change in the amplitude of dynamical signatures upon ligation can lead to observable allosteric differences, even in the absence of global conformational changes [3]. While comparative assessment of structure and function is a long-standing paradigm within proteins (e.g. [4], [5], [6]), comparisons of dynamics across orthologous proteins are rare because experiments are labor intensive and costly. In spite of these difficulties, the importance of such comparisons has resulted in a small number of experimental assessments [7], [8]. Similarly, in their seminal paper, Lee *et al.* used sidechain order parameters to assess the degree of conservation across three PDZ domains [9], which identified nontrivial conservation greater than expected by sequence similarity. Therein, they further conclude that sidechain dynamics are affected by nonlocal events, including correlated motions. Unfortunately, the technical difficulties of performing these experiments make comprehensive comparisons prohibitive.

Computational methods are promising alternatives to characterize and compare protein dynamics across protein families [10], [11], [12], [13], [14], [15]. In addition to being much less costly than experimental interrogations, computational methods are generally able to characterize protein backbone and sidechain dynamics in more detail than experimental means (depending upon the level of coarse-graining). Nevertheless, the computational expense associated with traditional simulations methods continues to make comprehensive analyses impractical [16]. To circumvent the cost of simulation, we have developed an ensemble-based Distance Constraint Model (DCM) [17], [18] that is based on a Gibbs ensemble of topological networks, where each network encompasses all atomic geometries that are accessible under the same set of local constraints. Efficient rigidity graph algorithms [19], [20], [21] characterize network flexibility when applied to a single network. The DCM then averages over the thermodynamic ensemble to characterize equilibrium properties. While the details can be found elsewhere [17], [18], [22], the process of ensemble averaging requires an accurate estimate of the free energy associated with each network, which is based on a free energy decomposition approach that explicitly takes into account nonadditivity within conformational entropy components [23], [24]. The output of the DCM provides quantified stability/flexibility relationships (QSFR) [25], [26], which is a high dimensional description of protein thermodynamics, dynamics and their interrelationships. In all works to date considering protein QSFR, we have employed a minimal DCM (mDCM) that considers hydrogen bonds (H-bonds) and native torsion forces as fluctuating interactions.

Much of our recent work has focused on development and application of methods for comparing QSFR across protein families. Across a mesophilic/thermophilic RNase H pair [26], four bacterial periplasmic binding proteins [27] and nine oxidized thioredoxins [28], our results reveal an intriguing mix of conservation and variation within protein flexibility, consistent with experimental trends. As one might expect based on fold conversation, we observe general conservation within backbone flexibility. Conversely, pairwise residue-to-residue couplings are highly sensitive to small protein differences. Going a step further, we have also recently developed a perturbation method that identifies allosteric sites based on changes to QSFR upon residue confinement that also revealed a nuanced mix of conservation and variation [29].

Using human c-type lysozyme as a model system, we now establish how much a single mutation affects protein flexibility. We analyze a dataset of 14 different point mutants that have been characterized under a narrow window of experimental conditions [30]. Somewhat surprisingly, we find that changes in flexibility upon mutation are very common. In fact, the number of positions with significant changes in flexibility characteristics is similar to the number of positions without change. Additionally, these changes can occur over relatively long distances, meaning they are frequently allosteric in nature. Changes that lead to increased backbone flexibility are slightly more common than changes that lead to increased rigidity. This asymmetry primarily occurs because many mutations lead to increased flexibility within lysozyme's β-subdomain. This result is noteworthy because several investigations have concluded that amyloid forming mutations lead to local unfolding in this region [31], [32], [33], [34], [35], which is the site of amyloid nucleation.

## Results

### Intrinsic Flexibility of Wild-Type Lysozyme

Lysozyme, which is abundant in egg whites and secretions, is a small (∼130 residues) globular enzyme that hydrolyzes cell wall β (1,4) glycosidic linkages. Human c-type lysozyme is a common model system for protein structure/function investigations because it is relatively easy to express and biophysically characterize. The dataset of lysozyme mutants considered in this report was constructed previously, where we used the mDCM to predict mutant melting temperatures with an average error of 4.3% [30]. Going further, the primary goal of this investigation is to critically evaluate the consequences of single point mutations on lysozyme flexibility. However, before doing so, we must first quantify wild-type lysozyme's intrinsic flexibility characteristics to be used as our reference point.

**Figure 1:**
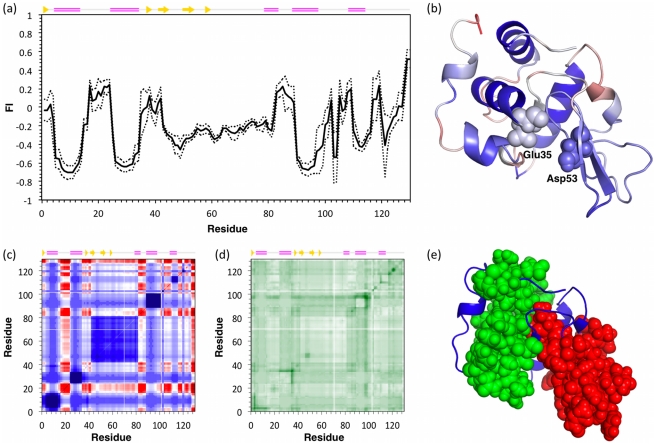
Intrinsic flexibility characteristics for lysozyme are shown. (**a**) The average flexibility index (FI) across a set of seven wild-type lysozyme structures is plotted versus residue number (solid line). The dashed lines indicate ±1 σ, which defines the noise range within the quantity. (**b**) Lysozyme is color-coded according to average FI values in panel (**a**), where red regions indicate flexibility (FI>0) and blue indicates rigidity (FI<0). (**c**) The cooperativity correlation profile of 2NWD identifies all pairwise mechanical couplings. Red indicates residue pairs within the same correlated motion, whereas blue indicates residues within the same rigid cluster. White indicates no mechanical coupling. Panel (**d**) shows the relative per pixel standard deviation across the wild-type set where darker color represents a greater value. There are two large rigid clusters identified in panel (**c**), which are highlighted in panel (**e**). The first (green) is defined by helices α1, α2, α4 and α5, whereas the second (red) corresponds to the β-subdomain. The active site is located at the cluster interface, and the hinge motion indicated in panel (**b**) allows the enzyme to close around its substrate.

We define an average flexibility profile using a set of 7 different human wild-type lysozyme structures. Therein, differences in flexibility solely arise from differences in the X-ray crystal structures. Moreover, the variability across the dataset establishes a baseline precision for the calculated properties. Values within ±1 standard deviation (±1 σ) from the mean of the wild-type set are taken to be within background noise, and are thus deemed equivalent. **Fig. 1a** plots the flexibility index (FI), which is an mDCM output that characterizes local flexibility. Positive values quantify flexible regions, whereas negative values quantify rigidity. Additionally, the variability within FI across the 7 wild-type structures is also shown. **Fig. 1b** maps the average flexibility profile to structure (blue = rigid, whereas red = flexible). In general, helices are mostly rigid, whereas spanning loop regions are mostly flexible. The β-subdomain is marginally rigid, with some interspersed flexibility. Lysozyme is composed of an α+β structure, where the β-subdomain is attached to the core via a known hinge region that is identified by the mDCM [18]. The flexible hinge region and lysozyme's two catalytic residues are also highlighted. Most of the other flexible regions correspond to loops connecting secondary structure elements.

A higher order description of protein dynamics is provided by cooperativity correlation (CC), which characterizes correlated motions and co-rigidity. Specifically, CC plots identify all pairwise residue-to-residue mechanical couplings. **Fig. 1c** plots the CC for the 2NWD structure, which is the closest to the geometric center of the wild-type set. Blue coloring identifies co-rigid residue pairs (meaning residue pairs with high probability of occurring within the same rigid cluster), whereas red coloring identifies flexibly correlated pairs (residue pairs within a correlated motion). Mechanically decoupled regions are colored white. The per-pixel variation across the wild-type set is plotted in **Fig. 1d**. Within **Fig. 1c**, two prominent rigid clusters can be identified. The first is composed of helices α1, α2, α4 and α5, whereas the second spans the β-subdomain region (cf. **Fig. 1e**). The active site and accompanying hinge motion corresponds to the cluster interface, which allows the enzyme to close around its carbohydrate substrate.

### Changes in Backbone Flexibility upon Mutation

**Figure 2:**
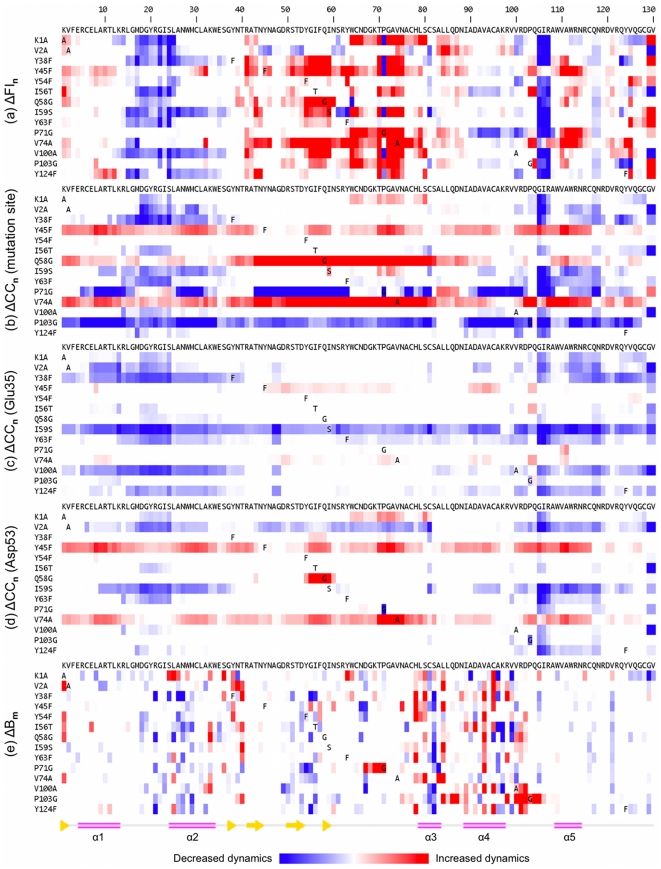
Comparison of backbone flexibility and changes across the dataset are shown. Panel (**a**) plots changes in ΔFI_n_ for each mutant relative to the wild-type structure. In the same manner, changes in cooperativity correlation (CC) with respect to the mutation site, Glu35 and Asp53 are respectively plotted in panels (**b**), (**c**) and (**d**). Panel (**e**) plots changes in the median normalized B-factors across the dataset.

The primary goal of this report is to investigate changes in lysozyme dynamics upon mutation. To that end, we analyze changes in FI and CC that occur upon mutation. The profiles defined above establish when a change in flexibility is significantly above background noise. That is, a change in flexibility is identified when the FI and/or CC value of a mutant position occurs beyond the ±1 σ cutoff, otherwise no change is said to occur. **Fig. 2a** plots the normalized change in FI (ΔFI_n_) for each mutant where red indicates increased flexibility, and blue indicates increased rigidity. Some common responses are identified regardless of the details of the mutation. Interestingly, flexibility increases frequently occur within the β-subdomain regardless of mutation position, while an increase in rigidity within the β-subdomain almost never occurs. Changes in the α -subdomain are slightly less frequent with the most common responses having increased rigidity within the α 1/α 2 loop and a 3-residue segment of the α 4/α 5 loop.

**Figure 3 pcbi-1002409-g003:**
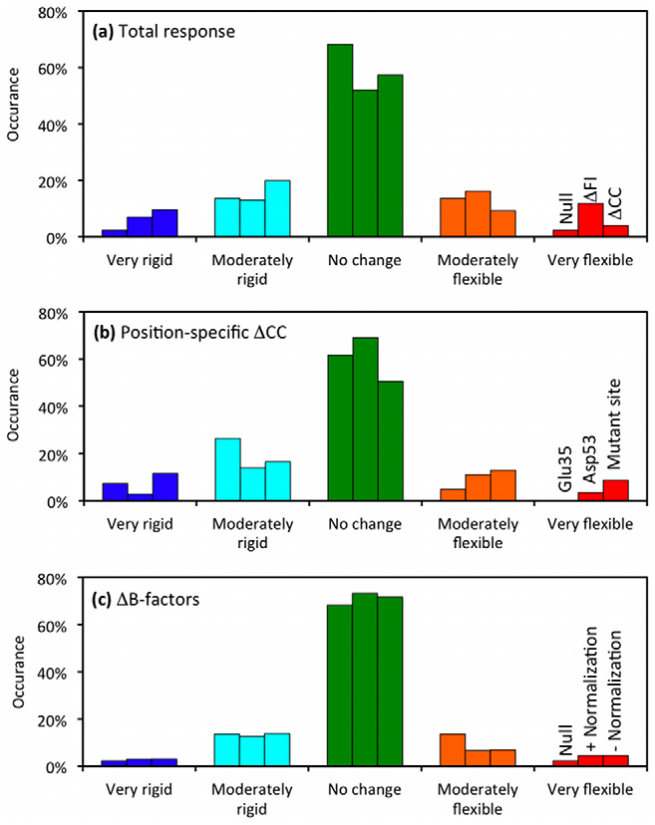
Flexibility response histograms are shown. Across a collapsed dataset constructed from all 14 mutant structures, each residue is binned based on changes to QSFR properties. The bins are color-coded by: green = no change, cyan and blue = moderate and large increases in rigidity, and orange and red = moderate and large increases in flexibility. In each panel, the bin order is conserved and indicated at the right. Panel (**a**) plots the null expectation histogram (highlighted with diagonal hashing) alongside the overall changes in flexibility index and cooperativity correlation. Panel (**b**) plots changes in cooperativity correlation with respect to specific residues: the mutation site, Glu35 and Asp53. Finally, panel (**c**) re-plots the null expectation alongside changes in B-factors (with and without median normalization).

Despite the above trends, many site-specific differences are obvious. Binning the Δ FI_n_ values across a collapsed dataset of all 14 mutants underscores this point. **Fig. 3a** indicates that the dynamics are appreciably changed in 48.0% of the residues upon mutation. Interestingly, the percentage of residues with increased flexibility (28.0%) is slightly more than the percentage with increased rigidity (20.0%). This result makes intuitive sense because all but one of the mutants decreases structural stability. We segregate moderate flexibility changes from large changes using a cutoff of ±2 σ. Percentages of large increases in flexibility are slightly more than large increases in rigidity (11.8 vs. 7.0%). Based on the ±1 σ definition of the “no change” background profile, the null expectation is that 68.2% of the positions should have “no change.” Further, moderate changes within 1 to 2 standard deviations, and large changes greater than 2 standard deviations, have null expectations of 13.6% and 2.3%, respectively. **Fig. 3a** clearly indicates that we observe more changes in FI than this random expectation. Using the chi-square statistic, the differences within the observed and random expected histograms are strongly significant (cf. **Table 1**). That is, changes in flexibility upon mutation are more common than the background variation across the set of wild-type structures.

**Table 1:** Statistical significance of the observed histograms.

Flexibility metric	p-value
ΔFI_n_	9.1E-212
ΔCC_n_ (all positions)	0.00
ΔCC_n_ (Glu35 only)	1.6E-122
ΔCC_n_ (Asp53 only)	2.7E-4
ΔCC_n_ (mutation site)	1.8E-237
ΔB_m_	2.6E-24
ΔB_r_	2.4E-22

Bin sizes within the expected histograms are defined from the variation across the set of wild-type structures: large changes >±2 σ, moderate changes are ±1–2 σ, and no change is between ±1 σ, from which background bin probabilities are calculated. The chi-square statistic is use to compare the expected and observed histograms, and the reported p-values quantify the probability that the histograms are equivalent. In all cases, the histograms are determined to be statistically distinct from the null expectation.

**Figure 4 pcbi-1002409-g004:**
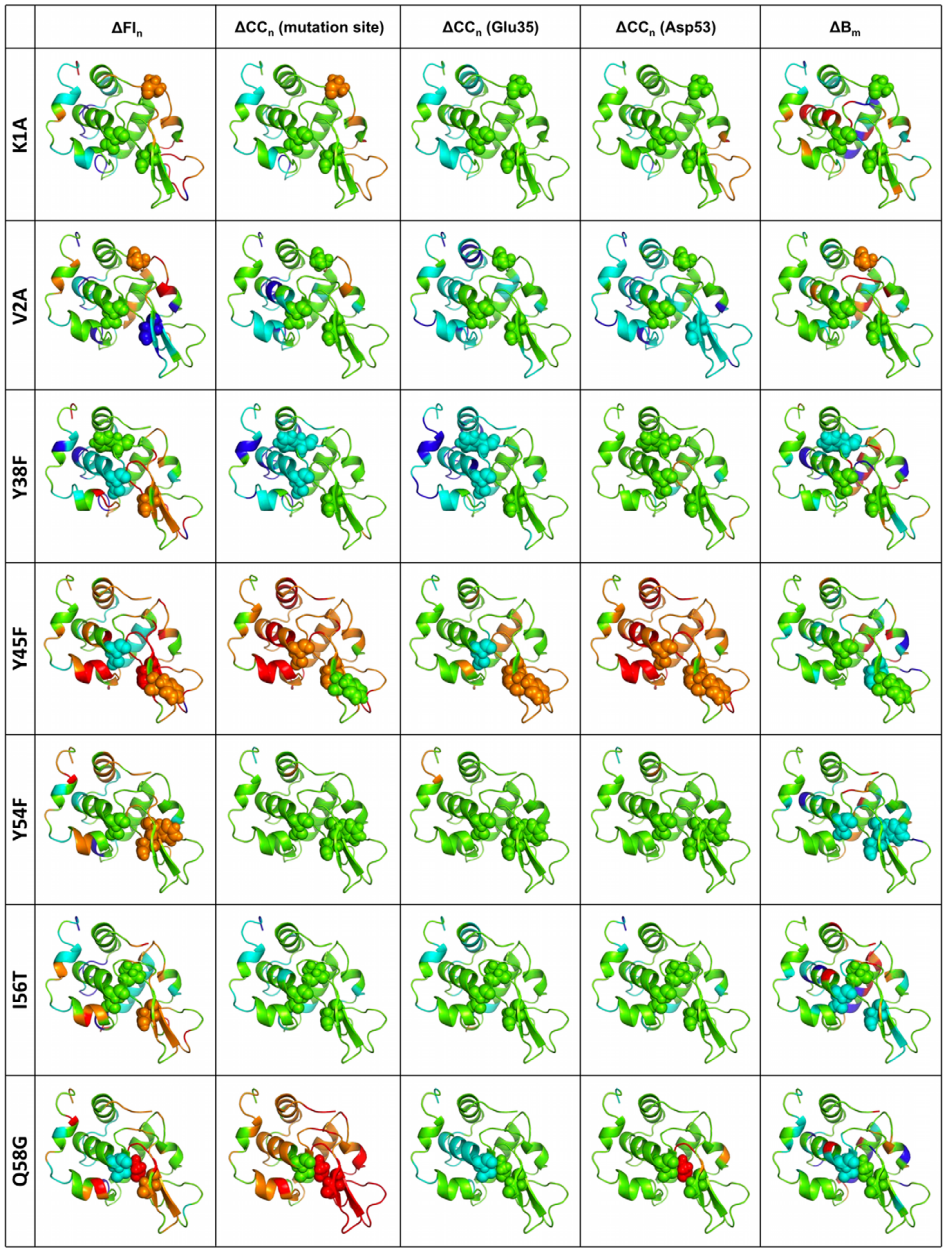
The affects of mutation on protein flexibility are mapped to structure. The five columns correspond to ΔFI_n_, ΔCC_n_ with respect to Glu35, ΔCC_n_ with respect to Asp53, ΔCC_n_ with respect to the mutation site, and ΔB_norm_. In all cases, the histogram bins in **Fig. 3** define the coloring schemes. The orientation of each protein is nearly identical across the figure. In each structure the catalytic pair (Glu35 and Asp53) and the mutated residue is rendered in spacefill. Importantly, this figure emphasizes the long-range nature of the response.

**Figure 5:**
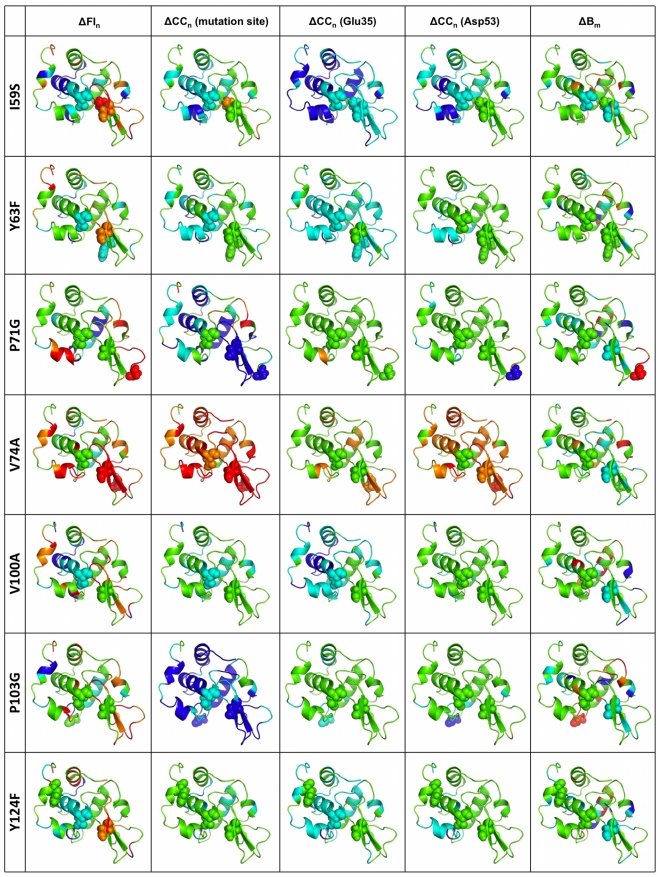
Continuation of **Fig. 4** using the same coloring scheme and structural orientation.

Using the same coarse-grained color scheme as **Fig. 3a**, the first column in **Figs. 4**–**5** color-codes the mutant lysozyme structures by Δ FI_n_ values. In each, the structures are shown in nearly identical orientations, and the mutated residue, Glu35 and Asp53 are rendered in spacefill view to orient the viewer. In addition to highlighting the frequency of changes in flexibility or rigidity upon mutation, this figure emphasizes that changes can be quite long-ranged. For example, the I59S mutation, which occurs within the β-subdomain portion of the active site cleft, affects the most distant portions of the structure. Even more pronounced is the P71G mutation. The mutation site is located on the outmost reach of the β-subdomain, yet it causes helix α 4 at the hinge and the α 4/α 5 loop within the main core of the protein to significantly rigidify. Concurrently, the β-subdomain and helix α 5 become much more flexible.

### Changes in Cooperativity Correlation upon Mutation

**Figure 6:**
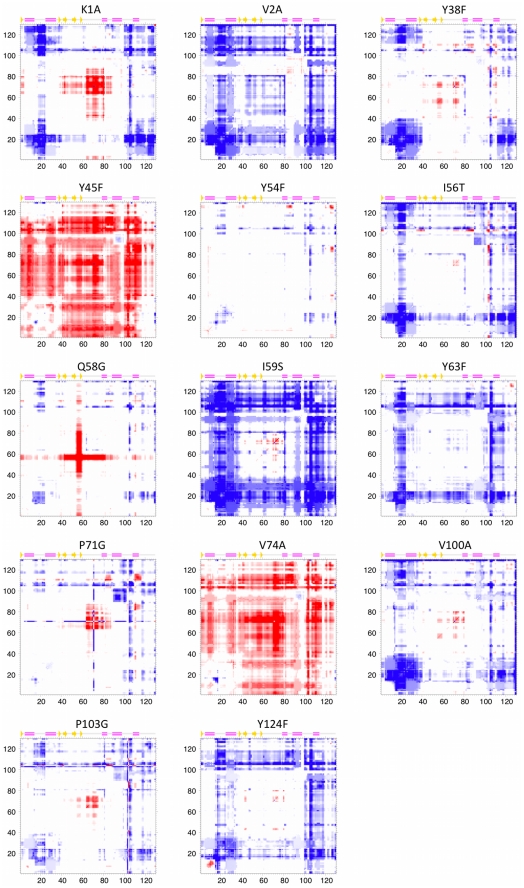
Cooperativity correlation (CC) difference plots show the differences in pairwise mechanical couplings between each mutant structure and the wild-type reference. Red indicates increased correlated flexibility within the mutant structure, whereas blue indicates increased correlated rigidity. Juxtaposed to the ΔFI results that show significant uniformity within their response, the ΔCC_n_ values are highly variable across the set of mutants.

Going further, **Fig. 6** shows the normalized changes in cooperativity correlation (ΔCC_n_) upon mutation, which reveals a much more rich and interesting set of changes in flexibility. Again, we characterize the degree of change with respect to the mean wild-type CC values using the same standard deviation ranges as above. Across all mutants, an increased correlated flexibility is observed in 42.7% of the CC values. Interestingly, the bias towards increased correlated flexibility observed in Δ FI_n_ is not present. Rather, Δ CC_n_ results are skewed in the opposite direction (cf. **Fig. 3a**). Specifically, increased rigidity correlation is observed in 29.5% of the Δ CC_n_ values, whereas only 13.2% have increased flexibility correlation. This asymmetry stresses the physical distinction between the two metrics. While the Δ FI_n_ results describe changes in backbone flexibility within a localized region, Δ CC_n_ identifies changes in pairwise mechanical couplings that uncover cooperative effects. The results from our dataset indicate that most of the increases in backbone flexibility are localized, frequently within the β-subdomain (with Y45F and V74A being the primary exceptions). Put otherwise, the local increases in flexibility identified by ΔFI_n_ are largely decoupled from other motions, which is why Δ CC_n_ does not show a large increase in correlated flexibility. Conversely, the increased rigidity correlation across the dataset indicates that most of the increases in backbone rigidity are frequently coupled to other rigid regions throughout structure.

As with ΔFI_n_, the differences in ΔCC and the null expectation are strongly significant (cf. **Table 1**). Another key deviation from the ΔFI_n_ results is the high variability across the set of mutants. For example, the Y54F mutant has little overall affect on the set of mechanical couplings within lysozyme. Conversely, the same mutation at position 45 leads to a large increase in flexibility correlation, whereas the Y→F mutations at positions 38, 63, and 124 slightly increase co-rigidity. A similar juxtaposition occurs within the V→A mutations. V74A drastically increases correlated flexibility; however, V2A has the opposite affect by drastically increasing correlated rigidity. While these cases represent nearly homogenous changes in CC, most of the remaining mutants have a mix of both increased correlated flexibility and correlated rigidity. Taken together, the large and diverse mutant-specific changes within the ΔCC_n_ results underscore the high sensitivity of the metric, which we have discussed previously [26], [27], [28].

It is technically difficult to exhaustively compare all changes because the two-dimensional nature of the data precludes linear descriptions along the lysozyme sequence. As such, we extract for further analysis strips of ΔCC_n_ values from the full plot for a single residue point of reference. Here, we examine ΔCC_n_ with respect to the mutation site and the two catalytic residues. These results are reported alongside the ΔFI values just discussed in **Figs. 2** **–** [Fig pcbi-1002409-g003] [Fig pcbi-1002409-g004] **5**, which underscores the richness within ΔCC_n_. For example, changes in CC with respect to Glu35 are common. Moreover, they can be quite large and frequently propagate over long distances. The same is true for ΔCC_n_ with respect to the mutation site. On the other hand, changes with respect to Asp53 are somewhat suppressed, yet still statistically significant. These cases emphasize that the extent and location of changes within the mechanical couplings is dependent upon the reference point. Similar types of differences are observed when examining ΔCC_n_ from other points of reference.

### Flexibility Is Distinct from Mobility

Protein dynamics can be quantified in many ways. Therefore, it is important to distinguish flexibility from mobility. From rigidity theory, flexibility indicates that a network is deformable, but it need not be mobile. For example, a stationary pivot of a swinging pendulum is highly flexible, but not mobile. On the other hand, the end of the pendulum can simultaneously be rigid and highly mobile [22]. Because of this physical distinction, it is useful to benchmark how mobility changes upon mutation. To that end, we compare changes in α-carbon atomic displacement parameters (B-factors) of each mutant structure to the wild-type profile. However, before doing so, it should be stressed that caution must be employed when analyzing B-factors in terms of mobility because protein crystals are not homogeneous. That is, protein structure B-factors reflect both temporal (i.e., mobility) and spatial disorder across the crystal lattice. B-factors are quantitatively affected by occupancies. Occupancies less than one can be an indication of disorder, but lead to improved R-factors [36]. As such, even when multiple structures have the same space group, direct comparisons of B-factors reflect substantially more than just differences in mobility. Thus, using B-factors to reflect mobility is only truly accurate when all other error sources have been removed. To help mitigate some of these caveats, we normalize B-factors using the median-based method of Smith et al. [37].

After normalization of the α-carbon B-factors within each structure, we calculate the wild-type background profile in the same way as above. Subsequently, normalized B-factors from each mutant structure are compared to the normalized wild-type profile using the same σ ranges as above in order to classify no change, compared to moderate and strong changes. Surprisingly, the histogram of median normalized B-factor changes (ΔB_m_) (**Fig. 3c**) is substantially different from the flexibility changes. Specifically, there are fewer changes in B-factors than one would expect based on the wild-type profile. This suppression of changes is statistically significant (cf. **Table 1**). Moreover, there is no correlation between the ΔFI_n_ quantities and ΔB_m_ values (results not shown), underscoring the differences between flexibility and mobility. Despite the cautionary note above about B-factor comparisons, we also compare the raw B-factor changes (ΔB_r_) to determine if normalization is biasing the results. **Fig. 3c** also shows that there are no appreciable differences between the ΔB_m_ and ΔB_r_ histograms. For completeness, the ΔB_m_ values are reported alongside the ΔFI_n_ and ΔCC_n_ results in **Figs. 2** **–** [Fig pcbi-1002409-g003] [Fig pcbi-1002409-g004] **5**. No correlation is found using raw data as well.

### Structural Considerations of the Flexibility Changes

**Table 2** counts the number of residue responses that occur for a given solvent accessibility and distance separation (mutation α-carbon to response α-carbon) range. The collapsed dataset of all residues is stratified by solvent accessibility for both the response (top) and mutation (bottom) sites. In each case, exposed, moderate, and buried respectively corresponds to the top, middle, and bottom thirds of all relative solvent accessibilities, which maintains similar observations in each stratum for the response and mutation sites. The ΔFI_n_ bins again correspond to those in **Fig. 3**. Interestingly, in both cases solvent accessibility has little effect on the response rate. In all cases but one, the ratio of changes to no change is approximately one. That is, a change in flexibility is generally as frequent as no significant change. Note that we focus on the ratio of changes because this normalizes out the size discrepancies — the strata corresponding to larger distances will naturally have bigger counts simply because there are fewer residues close to the mutation compared to farther away. The one noticeable exception to this general trend is when the mutant residue is solvent exposed, for which there is a significant decrease in flexibility changes. This relative lack of effectiveness in causing a change in flexibility makes intuitive sense because solvent exposed positions are naively expected to be more tolerant to mutation due to reduced steric constraints.

**Table 2:** Residue response statistics.

	Large rigidity increase	Moderate rigidity increase	No change	Moderate flexibility increase	Large flexibility increase	Ratio
*Distance from response site = 0 to 8 Å*						
Buried	3	7	26	7	17	1.31
Moderate	1	5	30	10	12	0.93
Exposed	1	3	21	8	12	1.14
**Union**	**5**	**15**	**77**	**25**	**41**	**1.12**
*Distance from response site = 8 to 16 Å*						
Buried	24	38	130	28	31	0.93
Moderate	9	31	130	35	27	0.79
Exposed	9	9	81	36	18	0.89
**Union**	**42**	**78**	**341**	**99**	**76**	**0.87**
*Distance from response site≥16 Å*						
Buried	25	41	155	53	23	0.92
Moderate	23	49	174	49	29	0.86
Exposed	32	54	200	68	46	1.00
**Union**	**80**	**144**	**529**	**170**	**98**	**0.93**
*Structural characterization of response site*						
Helix	95	170	553	117	45	0.77
Strand	1	4	51	30	26	1.20
Coil	31	63	343	147	144	1.12
α-Subdomain	115	220	693	177	69	0.84
β-Subdomain	12	17	254	117	146	1.15
*Mutant residue is buried*						
0–8 Å	4	9	33	9	16	1.15
8–16 Å	32	52	145	41	25	1.03
≥16 Å	28	37	153	41	25	0.86
**Union**	**64**	**98**	**331**	**91**	**66**	**0.96**
*Mutant residue moderately exposed*						
0–8 Å	0	4	23	14	17	1.52
8–16 Å	5	18	108	39	40	0.94
≥16 Å	22	58	175	78	49	1.18
**Union**	**27**	**80**	**306**	**131**	**106**	**1.12**
*Mutant residue is exposed*						
0–8 Å	1	2	21	2	8	0.62
8–16 Å	5	8	88	19	11	0.49
≥16 Å	30	49	201	51	24	0.77
**Union**	**36**	**59**	**310**	**72**	**43**	**0.68**
*Structural characterization of mutant residue*						
Helix	25	40	133	33	29	0.96
Strand	28	45	180	84	53	1.17
Coil	74	152	634	177	133	0.85
α-Subdomain	65	120	408	108	79	0.91
β-Subdomain	62	117	539	186	136	0.93

Each cell counts the number of residue responses (ΔFI) that correspond to a given solvent accessibility range (or structural element) for a given distance to the mutation site. The collapsed dataset of all residues is stratified by response residue solvent accessibility in the top half of the table, whereas the collapsed dataset is stratified by mutation site solvent accessibility in the bottom half. The ratio value in the last column is the number of residues with altered flexibility divided by the number of residues with no change.

**Table 2** additionally provides statistics comparing structural features of the response and mutation sites. First, the dataset is stratified by secondary structure. As discussed above, there is a slight reduction in the relative response rate for α-helical positions. Conversely, there is slight increase in the β-strand positions, which is strongly skewed towards increases in flexibility. **Table 2** also provides statistics for the α- and β-subdomains, which parallels the secondary structure results. That is, the β-subdomain is highly susceptible to increased flexibility upon mutation. Conversely, a mix of changes in the α-subdomain commonly occurs, albeit at a rate slightly lower than no change. Interestingly, the ratios are more similar (∼1) across secondary structure and subdomain boundaries when focusing on the mutation site, with coil residues being the sole exception. Mutation of coil residues tends to have a decrease in the relative response rate, which simply reflects the same observation above for mutation of solvent exposed residues. The ratios for ΔCC_n_ are qualitatively similar, albeit slightly less across the entire dataset. The average ratio for ΔCC_n_ is ∼0.7, meaning a lack of change in CC is more common than a change. Nevertheless, changes in CC that have been observed as general trends in prior work [26], [27], [28] are observed here as evident in most cases within **Fig. 6**, where drastic changes usually appear within a small number of strips. However, there are certain cases (i.e., V2A, Y45F, and V74A) where virtually the whole CC plot is affected.

## Discussion

### Changes in Flexibility upon Mutation Are Common and Large

In previous reports, we have investigated how familial divergence affects protein dynamics and, as a consequence, allostery. Our initial work along these lines compared a mesophilic and thermophilic RNase H pair [26], which reproduced experimental conclusions regarding the balance between molecular flexibility and thermodynamic stability [38], [39], [40], [41]. Subsequently, we expanded our comparisons to 4 bacterial periplasmic binding homologs [27] and 9 oxidized thioredoxin structures [28]. Taken together, our collective results suggest an intriguing mix of conservation and variability within stability and flexibility. Pairwise mechanical couplings that provide a higher order description of flexibility and rigidity are generally sensitive to small differences. The latter result highlights how small structural variations are amplified into global differences as mechanical couplings propagate through the network.

In addition, we have linked mechanical and thermodynamic response to allostery, where a perturbation method is used to identify putative allosteric sites [29]. Therein, we introduce a small number of constraints to mimic the effect of ligand binding, from which new QSFR properties are calculated using the same structure. Large changes in QSFR metrics indicate an allosteric response. Application of this method to 3 CheY orthologs indicates that the most conserved response occurs within the β4/α4 loop, which is known to be important to propagation of the CheY phosphorylation signal [42], [43], yet residue-level response is quite variable, leading to the conclusion that allosteric response is both variable and conserved across the CheY family. The variability in ΔCC observed above further demonstrates diversity and sensitivity of allosteric response, which is consistent with observed variations within allosteric response across protein families (cf. [44] and references therein).

The ubiquity of differences observed across sets of orthologous proteins, which is consistent with myriad experimental results, leads one to wonder about the origins of the familial divergence. That is, how many mutations are needed to observe significant differences in protein dynamics? As such, using human c-type lysozyme as a model system, this paper quantitatively assesses the differences in protein flexibility that occur upon individual point mutations. In spite of the rather small structure differences, it is common for changes in flexibility to occur throughout structure, including at locations remote from the mutation site. As indicated by the histograms in **Fig. 3a**, changes in both flexibility metrics are common. Specifically, while no change is the most frequent response, 42–48% of the residues undergo an appreciable change upon mutation. These distributions are obtained by sampling a collapsed dataset composed of all residues for each protein in the dataset (or as a variant to the method, across the entire protein except for a local window centered on the mutation site). This means that it is not the case that one particular mutant will make virtually no change, whereas another will make a large change. Rather, a typical mutant includes many sites with increased flexibility and increased rigidity throughout the protein. Exactly where the changes occur has a great variance in general, but the statistical expectation of having compensation between one part of the protein increasing in rigidity while another part of the protein increases in flexibility seems very consistent across our dataset. The percentage of positions leading to increased backbone flexibility (27.9%) is slightly greater than the percentage increasing rigidity (20.0%). In summary, *changes in backbone flexibility upon mutation are common*, where local changes across the protein are typically composed of comparable amounts of an increase and decrease in flexibility distributed throughout the protein. Essentially, the protein is maintaining a global level of marginal mechanical stability within the native state at the melting temperature of the mutant. Changes in CC are also common; however, the differences between increased flexibility and increased rigidity are more asymmetrical. As discussed above, it is found that flexibility increases upon mutation tend to be localized, whereas increases in rigidity are likely to be coupled to remote structural sites. This result is not a matter of simple statistical chance that as more regions become rigid, the tendencies of these regions to coalesce into larger rigid regions increase. Rather, the increase in co-rigidity is counter-intuitive based on this reasoning, since there is an overall decrease in rigidity across the protein upon most mutations. This simultaneous effect suggests sparse and ramified rigid pathways are carved out by the mutations, which is critical to maintain marginal mechanical stability within the protein at its melting temperature. Here, critical means that further degradation of this pathway is likely to lead to unfolding as rigidity in the protein is lost [45].

**Figure 7:**
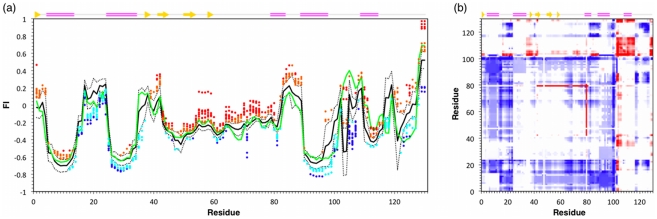
Mutational affects on flexibility. (**a**) Lysozyme backbone dynamics are characterized by a flexibility index (FI). Positive FI values measure flexibility, whereas negative values measure rigidity. The structure is isostatically (marginally) rigid when FI = 0. The black solid line indicates the average human wild-type lysozyme profile, whereas the dashed lines indicate ±1 σ. The mutant sites that moderately score beyond the background are indicated using the same coloring scheme as **Fig. 3**. The green solid line indicates hen egg white lysozyme backbone flexibility (HEWL), which is generally more similar to the wild-type profile than the human mutants. (**b**) The difference between human wild-type lysozyme and HEWL cooperativity correlation is shown. The coloring scheme is the same as in **Fig. 5**.

To further support the conclusion that changes in flexibility upon mutation are common, we also assess the flexibility differences between human wild-type and hen egg white lysozyme (HEWL). **Fig. 7a** compares changes in HEWL backbone flexibility (relative to human wild-type) to the mutant changes summarized above. Surprisingly, the number of differences between the two orthologs is generally slightly less than observed within the mutant dataset. While, on average, 48.0% of the mutant positions have a change in FI, only 41.1% of the HEWL positions changes. Although there is relative decrease in number of flexibility differences, the number of changes that do occur is statistically significant (p = 2.0E-7). Moreover, the scale of the ΔFI values for HEWL falls within the variation across the human mutant dataset despite the fact that the pairwise sequence identity is only 61%. That is, even with a significantly reduced sequence identity, there are no wholesale differences in flexibility. Put otherwise, the changes in backbone flexibility within the mutant structures are clearly large since they are on the same scale as the much more divergent HEWL ortholog. Similarly, the HEWL ΔCC_n_ results (**Fig. 7b**) are also easily within the mutant dataset range established in **Fig. 6**.

It is worth noting that our dataset composition is inherently biased towards rigidity. That is, the studied mutations are all amendable to crystallography, which eliminates many possible mutations that destabilize the structure so much that it is too flexible to form a crystal lattice. As such, our conclusions regarding the frequency and extent of flexibility changes would be even greater if it were feasible for us to study all possible mutations because extreme increases in flexibility upon mutation are actually underrepresented in our dataset.

### Changes in Flexibility Can Be Long-Ranged

We have segregated responses into moderate and large changes (cf. **Fig. 3**). As expected, moderate changes are the most common, but large changes in FI and CC also occur frequently (respectively, 18.3 and 13.5% of the time). While the definition distinguishing between moderate and large is somewhat arbitrary, the ubiquity of large changes is clearly shown in **Fig. 2**. Moreover, large changes in backbone flexibility can occur anywhere in structure, but some clustering is evident. Specifically, large increases in rigidity are more likely to occur within the α1/α2 and α4/α5 loops, whereas large increases in flexibility tend to occur within the β-subdomain. Conversely, there is little clustering of CC response. These two opposing observations further emphasize our previous results that FI is strongly related to overall structural topology, whereas CC is highly sensitive to small differences within the H-bond network [27].

The visual survey of the first column in **Figs. 4**–**5** shows that changes in flexibility are rarely localized around the mutation site, but rather generally propagates over long distances. This observation is confirmed by the counts in **Table 2**. However, skewness in raw counts can be expected by the increased number of sites that are present in the strata corresponding to larger distances. Interestingly, the ratio of changes to no change for short, medium and long distances are all nearly equal to one (with the two exceptions explained above in the results section). The similarity in the ratios is somewhat surprising because the naïve expectation is that short-range changes would be much greater than long-ranged due to dampening effects. As such, these results indicate that *changes in protein flexibility upon mutation can be long-ranged*. Upon further statistical analysis, it is found that the ratios are not regimentally affected by solvent accessibility of the mutation or response site. In addition, the distance between the mutation-response pair has no systematic affect, meaning that neither structural distance nor solvent accessibility has a large biasing affect on the results. The sole exception being that mutations at solvent exposed positions is less likely to lead to changes in flexibility. Note that there are insufficient data to perform a statistically significant two-dimensional stratification that considers both response residue and mutant accessibilities.

### Relating Computational and Experimental Observations

Our results collectively indicate that point mutants cause a rich and diverse set of flexibility changes throughout structure. Generally, changes in both flexibility and rigidity within the protein upon mutation occur concurrently to maintain marginal mechanical stability at the new melting temperature. Many changes are localized, but significant portions propagate over surprisingly long distances. While we cannot make a direct quantitative comparison to experimental results because the observed response properties are fundamentally distinct, changes in NMR order parameters show similar response richness. For example, many reports have used N-H S^2^ order parameters to demonstrate that changes in backbone dynamics can be quite large upon mutation (e.g., see [46], [47], [48], [49], [50]), yet the magnitude of the changes are generally within the scale wild-type order parameter distributions [51]. The observed changes in backbone flexibility are qualitatively equivalent (cf. **Fig. 7**). Moreover, localized increases in dynamics have been observed despite globally similar average structures [52] and stabilities [53] between the wild-type and mutant proteins. Particularly noteworthy are experimental results that mirror the complexity that we uncover on lysozyme on two additional small model-system proteins. First, concurrent increases in dynamics and rigidity have been demonstrated in the V54A Eglin c mutant [54], which epitomizes the changes in lysozyme flexibility within in **Figs. 2** and **4**–**5**. Second, long-ranged changes in dynamics have been observed within the F22L and A20V mutants of protein L [55], which is again shown for changes in lysozyme flexibility in **Table 2**.

Methyl sidechain S^2^ order parameters characterize ps-ns timescales, whereas backbone S^2^ order parameters characterize slower motions. While the DCM does not model dynamical timescales per se, experimental investigations that probe both further underscore the complexity and long-range nature of changes in protein dynamics upon mutation. For example, Igumenova et al. demonstrated that calmodulin backbone dynamics are largely unchanged upon mutation [56]. However, sidechain motions are significantly altered by the D58N mutation in the Ca^+^-binding loop, which are spread over long distances. Interestingly, the pseudosymmetric D95N mutation has no appreciable affect on sidechain dynamics. Similarly, Clarkson and Lee characterized two valine-to-alanine eglin c mutants [57]. Large dynamical changes were observed as much as 13 Å from the mutation site. The V54A actually causes a network of residues to increase in rigidity despite the fact that the mutation is thermodynamically destabilizing. Changes in the V14A mutant, which is also buried in the core of the protein, were much less. This diversity of response led the authors to conclude, “*…dynamical responses will be context-dependent,*” which is epitomized by our lysozyme dataset. That is, the affects of mutation are quite varied and highly dependent upon the local details of the perturbation, which propagate in complex and unexpected ways.

The Dobson lab has characterized dynamical changes in lysozyme, with a special focus on mutant amyloidogenicity. In particular, changes in I56T and D67H were studied using hydrogen/deuterium exchange NMR and mass spectrometry [58]. (Note that the I56T mutation is included within our dataset.) They showed that β-subdomain dynamics in the D67H mutant are changed extensively, whereas changes occur much less in the I56T mutant. This result broadly agrees with our results, which indicate that I56T dynamics are changed much less than mutants with the biggest responses (e.g., Y45F, I59S, V74A, and V100A). Taken together, our conclusions are therefore in line with many experimental characterizations of changes in protein dynamics upon mutation.

### Amyloid Formation and the β-Subdomain

Based on our previous investigations, we believe the above results could be generalized to most globular proteins. In addition, our results also reveal an interesting effect specific to lysozyme. That is, a large number of mutations, regardless of location or type, cause increased flexibility within the β-subdomain, which in many cases can be thought of as local unfolding. This point is noteworthy for two reasons. First, this result again highlights the long-range nature of dynamical changes because many of the mutations occur outside of the β-subdomain. Second, several experimental reports have suggested that mutations leading to amyloid in lysozymes and the related α-lactalbumins are due to structural changes, which may include local unfolding, in the β-subdomain [31], [32], [33], [34], [35]. As such, the partially unfolded β-subdomain may serve as a nucleation site for amyloid growth. Of course, our results do not address this issue, but they do parallel the earlier experimental conclusions. For example, ΔFI clearly indicate that the amyloidogenic I56T mutation has increased flexibility within β-subdomain (cf. **Figs. 4** **–** **5**). Similarly, our results indicate that several other mutants display at least as much flexibility therein, including K1A, Y38F, Y45F, Q58G, I59S, P71G, V74A, V100A and P103G. As such, it is tantalizing to consider that they might also be amyloidogenic. We have searched the literature and, to the best of our knowledge, these mutants have not been characterized. We therefore present them as blind predictions, and hope that others will consider characterizing their amyloidogenicity.

### Relating the Observed Changes to Protein Family Evolution

Across the dataset, changes in protein flexibility upon mutation are common, large and can be long-ranged. That is, the stark variation in dynamics observed across protein families unexpectedly occurs early in the divergence process through a combination of flexibility increases and decreases. However, the observed changes seldom significantly alter global flexibility. The relative similarity in positive and negative ΔFI_n_ values suggest that as divergence occurs, marginal mechanical stability is generally maintained because only incremental overall changes will be typically encountered by any given mutation. In other words, a single mutation will typically not overwhelmingly rigidify the protein nor overwhelmingly increase flexibility. Rather, structure subtly rearranges in response to the mutation to maximize enthalpy-entropy compensation. That is, a global increase in rigidity creates a large reduction of conformational entropy that is unfavorable, and a global increase in flexibility creates a large loss in enthalpy (weakened native contacts) that is unfavorable. Thus, the native state ensemble of the protein seeks to find the lowest free energy that typically requires a balance between flexible and rigid structural regions, suggesting that a mixture of rigidity and flexibility is typical at physiological conditions.

These results suggest that global increases in rigidity or flexibility upon mutation are rare because the local responses are derived in the noise (random fluctuations) around overall being neutral, with only a slight advantage towards increased flexibility in this case. The implication of the above is that successive mutations during the evolutionary process are generally necessary to substantially alter global flexibility characteristics. Viewed from a dynamics point of view (excluding selection in maintaining function), the process is a random walk capable of nudging the protein towards global increases in rigidity or flexibility. However, conservation of function is likely to select against systematic drift that leads to large differences in flexibility with respect to the function and stability of the wild-type protein. In that vein, the suppressed flexibility differences observed in HEWL actually suggest that additional compensating mutations can reestablish desired dynamical properties. For example, the similarity between human wild-type and HEWL β-subdomain flexibility is very persuasive given how susceptible this region appears to be to increased flexibility within the point mutants. This may, in part, explain why our prior results have shown backbone flexibility to be so well conserved across protein families.

On the other hand, our prior works also establish that CC is generally varied across a protein family due to differences in the underlying H-bond network [27]. Nevertheless, it appears that wholesale differences are not tolerated across protein families. The changes observed in **Fig. 6** indicate that a single mutation is sufficient to significantly alter global CC properties, where the accumulative effect of a few mutations should be sufficient to go beyond the range of differences we have observed across protein families. As successive mutations appear, conservation of function again provides the selection bias for proteins to maintain globally similar dynamics while evolving to varying stability characteristics. This scenario explains the considerable diversity in detailed dynamical changes occurring from a single point mutation, while general statistical characteristics remain robust.

### Conclusions

In this report we demonstrate that changes in human c-type lysozyme flexibility upon mutation are frequent, large, and can be long-ranged. Depending upon metric tracked, residue-specific flexibility is changed 42–48% of the time across the dataset. The mutation-induced structural perturbations propagate over long distances. In fact, the average distance between the mutant and affected residue is 17–20 Å. While direct quantitative comparisons to experiment are impossible due to different physical response characteristics studied and lack of experimental characterizations on most of the dataset, the frequency, scale and complexity that we find in flexibility changes are principally consistent with multiple NMR characterizations of mutant dynamics in a variety of proteins, including lysozyme. Intriguingly, we have shown that changes in flexibility upon single site mutation are generally larger than differences between hen egg white lysozyme (HEWL) ortholog to the human wild-type. In particular, most mutants lead to increased β-subdomain flexibility; however, β-subdomain flexibility within the human and HEWL ortholog remains conserved. Based on a random selection of mutations, this result is highly improbable because the human and HEWL lysozymes only have 61% sequence identity. As such, we hypothesize that evolutionary compensating mutations in HEWL have reestablished desired properties.

## Methods

### The Distance Constraint Model

Network rigidity graph algorithms are commonly used to study protein stability and dynamics [20], [45], [59], [60], [61], [62], [63], [64]. Therein, a topological framework (graph) is used to describe a set of geometric conformations. Atomic locations are described as vertices and chemical interactions are modeled as distance constraints (edges) that fix the relative position between atom pairs. From an input framework, pebble game (PG) algorithms quickly identify mechanical properties of the network. Starting from a completely disconnected graph (no edges), all vertices are assigned 6 “pebbles,” corresponding to the 6 trivial degrees of freedom (DOF) of a rigid body. Distance constraints are recursively added to the network, and pebbles are used to “cover” each *independent* constraint. That is, constraints that restrict the internal DOF are identified by the ability to remove an internal DOF. Frequently, especially as the PG progresses, pebbles are not immediately present within the considered atom pair due to the presence of other distance constraints on one or both of the vertices. In these cases, a pebble search is launched in attempt to collect free pebbles from remote locations. If pebbles can be found elsewhere, then the distance constraint is covered in the same way as before. If not, the constraint is said to be *redundant* and has no effect of the internal DOF of the network because the constraint has been placed in an already rigid region of the network. This process is repeated till all constraints have been added. Once the PG is complete, all rigid and flexible regions within the network can be identified.

While the computational speed of the PG is attractive, the approach is limited by its athermal formulation. That is, fluctuations within the presence of chemical interactions are not modeled. While this blunt approach is suitable in some situations (i.e., glass systems [21], [65], [66], [67], [68]), it is clearly problematic in protein structures where noncovalent interactions continually break and reform. To that end, the DCM was developed as a statistical mechanical model that introduces fluctuations into the network rigidity paradigm. Specifically, the DCM considers a Gibbs ensemble of network rigidity frameworks, each appropriately weighted based on its free energy. The free energy of each framework is calculated using free energy decomposition (FED). That is, each constraint is associated with a component enthalpy and entropy, and the total enthalpy of a given framework is simply the sum over the set of distance constraints. However, entropy components are nonadditive due to correlations within the dynamics, thus simple sums result in drastic overestimations of the total entropy. Entropy components are additive only over the set of independent DOF [23], [24]. As such, the DCM uses the PG to restore the utility of FED by summing entropy components only over the set of independent constraints. Introducing distance constraints based on their order of entropy (from smallest to largest) provides a rigorous lowest upper bound estimate of the total entropy [17].

Covalent bonds are quenched, meaning they are ever present, thus they do not need to be parameterized since the set is uniform across the ensemble. Conversely, topological differences arise due to fluctuating noncovalent interactions. For example, H-bonds can be present or not (salt bridges are modeled as a special case of H-bonds). Treating each interaction as independent, the number of different frameworks in the ensemble that would arise solely from H-bonds is 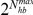, where 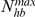 is the maximum number of H-bonds. The basin depth and amount of accessible phase space associated with each interaction type are respectively given by the enthalpy and entropy parameters (our convention is that enthalpic parameters are given Roman characters, whereas entropies are assigned Greek). In the mDCM, two types of fluctuating interactions are considered: H-bonds and torsion angle forces. The enthalpy of each H-bond (*u_hb_*) is calculated based on local geometrical considerations within the input structure using a modified [29] empirical potential [69], whereas its entropy (γ*_hb_*) is assumed and parameterized to be a linear function of its energy. For simplicity, torsion forces are segregated into native and disordered states, where the enthalpy and entropy of the native state is less than the disordered (*v_nat_*<*v_dis_* and δ*_nat_*<δ*_dis_*).

A maximal graph is identified from the input structure where all 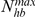 possible H-bonds and *N_tor_* possible torsion forces are identified. The mDCM ensemble is then constructed by perturbing away from the maximal graph. The number of frameworks within the ensemble is astronomical (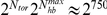
for lysozyme). As such, the partition function cannot be exhaustively summed. In response, the process of solving the mDCM for proteins is based on heterogeneous mean field theory [17]. A free energy landscape is defined by order parameters that specify the number of H-bonds (*N_hb_*) and native torsions (*N_nat_*) within a given macrostate. Within the macrostate, several hundred frameworks that satisfy the macrostate (*N_nat_*, *N_hb_*) are sampled using Monte Carlo, from which average properties are calculated. The free energy of a given macrostate is given by the free energy functional:
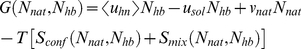
(1)Most of the variables in Eq. 1 have already been defined, with the exception of *u_sol_*. When a H-bond breaks, there is an enthalpic compensating interaction with solvent that is described by *u_sol_*. The mixing entropy term, *S_mix_*, arises from the various combinations that can satisfy the order parameters. The total conformational entropy, *S_conf_*, is appropriately attenuated by the probability of a distance constraint to be independent:
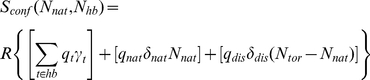
(2)That is, the PG algorithm is applied to each sampled framework in order to identify the set of independent and redundant constraints. From which, the conditional probability, *q_i_*, for constraint *i* to be independent when present is determined. The first term in the equation is a sum over all H-bond constraints due to large heterogeneity within their strength. The way Eq. 2 is written, *q_nat_* is equal to fraction of native torsion constraints identified as independent using the PG algorithm, and *q_dis_* is the fraction of disordered torsion constraints identified as independent. The values of *v_dis_*, δ*_dis_* and the empirical linear relationship between γ*_t_* and energy have been fixed in prior works [18], whereas *u_sol_*, *v_nat_*, and δ*_nat_* are fitting parameters. Note that the DCM would revert back to an additive FED scheme if the *q_i_* conditional probabilities were not present in Eq. 2.

The 2D free energy landscape is calculated over a grid to include all possible values of the order parameters, (*N_nat_*, *N_hb_*) that control the number of native torsions and number of H-bonds present in the protein. For temperatures near the melting temperature, two free energy basins separated by a saddle form. The basin that has greater numbers for *N_nat_* and *N_hb_* correspond to the native state. Conversely, the basin that has smaller numbers for *N_nat_* and *N_hb_* is associated with the unfolded state. At the melting temperature, 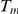, defined where the heat capacity is a maximum, the lowest free energy in the native basin is given by 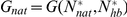 where 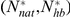 locates the specific point on the grid where the free energy is a minimum in the basin. At any grid point, an ensemble of constraint topologies can be generated because the probability for each type of constraint is known from the process of solving the free energy functional in Eq. (1) as previously detailed [17]. Then, for the mechanical property, 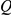, an ensemble average over many networks is made, which is denoted as 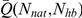. Then, the full average over the native basin is given by:
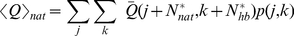
(3)where the sum over {j,k} is over a local neighborhood around (j = 0, k = 0) corresponding to the minimum point of the native basin. Beyond a certain range in the 2D free energy landscape, the probability 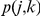 is negligible. In all the proteins studied here, a well-defined local neighborhood is found that is confined to a region before the saddle is reached. In other words, two state folding is observed. The probability function 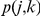 is normalized over the neighborhood that spans the native basin, and is given as:
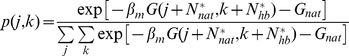
(4)where 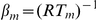 is the inverse thermal energy at the melting temperature. The probability 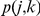 is the Gibbs probability for a protein in its native state to be in a specific macrostate making up the native basin at the melting temperature.

### Flexibility Index and Cooperativity Correlation

The flexibility index (FI) and cooperativity correlation (CC) are ensemble-averaged quantities over the native basin in the free energy landscape at the melting temperature. For a given macrostate, a sample constraint network is constructed using the probabilities for individual constraints to be present as described previously [17]. When no native torsions are present and no H-bonds are present, all the rotatable-bonds in the network are labeled from 1 to N. As constraints are added to the network, some of these bonds will become part of rigid regions. Then, for a given constraint network, a rigidity analysis is performed, and each *a priori* rotatable bond is identified as being: (*i.*) flexible because it is part of an under-constrained region, (*ii.*) locked because it is part of an isostatically rigid region, or (*iii.*) locked because it is within an over-constrained region. These three types of regions define clusters within the protein. No other possibility can occur [66], and all rotatable bonds are assigned to 1, and only 1, cluster. If the cluster is over-constrained, this means there are more constraints in the region than is necessary to make it rigid. If the cluster is isostatic, then the region is rigid, but there are just enough constraints to make it rigid. If there are not enough constraints within a certain region, it will be flexible.

Each bond is assigned a flexibility index, *f_i_*, that is defined based on a single constraint network as follows. If the bond in question is part of an isostatically rigid region, *f_i_* = 0. If the bond in question is part of a flexible region, the number of rotatable bonds within that flexible region is counted, and is denoted as *H*. The number of independent disordered torsions within that same flexible region is counted, and is denoted as *A*. To represent the density of independent DOF within the flexible region, the value *f_i_ = A/H* is assigned to all bonds within this cluster. Finally, if the bond in question is found to be in an over-constrained region, the total number of *a priori* rotatable bonds are counted, and denoted as *D*. Furthermore, the total number of redundant constraints within that region are counted, and is denoted as *B*. The value *f_i_ = B/D* represents the density of redundant constraints within this over-constrained region, and it is assigned to all the bonds within this cluster. Once this counting is complete for every cluster, every *a priori* rotatable bond in the protein will have a flexibility index assigned to it. To distinguish between densities of DOF versus redundant constraints, the *f_i_* values corresponding to flexible regions are positive, whereas the above *f_i_* values in over-constrained regions are multiplied by −1. We focus our analysis herein on just the backbone *a priori* rotatable bonds that comprise the ϕ and ψ angles of all residues (except proline, for which there is just a ψ angle).

In the final stages of the process, we typically average over 1000 or more realizations to obtain averaged mechanical properties for a given macrostate, (*j,k*). Then, for the *i*-th *a priori* rotatable bond, we have 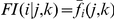, where the bar is used to indicate an arithmetic mean over all samples randomly generated by Monte Carlo sampling subjected to the given macrostate (*j,k*). The reported FI for the *i*-th *a priori* rotatable bond is given as: 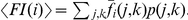


We employ a similar procedure to calculate the average value of CC. The main difference is that CC represents a pair correlation so the end result is a symmetric square matrix rather than a one-dimensional array. The variable 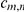 is equal to *f_m_* if the *m*-th and *n*-th *a priori* rotatable bonds are simultaneously found to be in the same flexible, isostaticaly rigid or over-constrained region. This is because the same value is assigned to all *a priori* rotatable bonds within a given cluster type. The correlation becomes apparent whenever two distinct types of clusters are identified. For example, if the *m*-th and *n*-th rotatable bonds are both found to be in rigid clusters, but these clusters are distinct, then 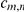 is equal to 0. In general, 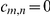 if the *m*-th and *n*-th *a priori* rotatable bonds belong to distinct clusters (whether of the same type or not). Thus, it should be noticed that no distinction is made between two *a priori* rotatable bonds being simultaneously found in the same isostatic rigid cluster versus in two different rigid clusters. It turns out that the relative frequency of two bonds being in an isostatic rigid region is very low. The distinction for why 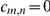 was initially a concern, and different measures have been considered. However, it was found that the reported average CC plots provide ample information regarding how flexibility and rigidity propagate through a protein [17], [18], [25], [26], [27], [28], [29]. We prefer to use the CC plot based on the density information as described here because it directly connects to the FI. In the next stages of the calculations, 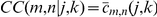 is the conditional average for a given macrostate, and the reported CC is given as 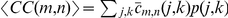. Using this procedure, CC plots identify all pairwise residue-to-residue couplings across the structure (cf. **Fig. 1c**). Consequently, correlated motions associated with a high density of DOF show up in red, while a high density of redundant constraints show up in blue. Regions that are marginally mechanically stable or simply uncoupled show up as white.

### Assessing Changes in Flexibility

Perhaps the most critical aspect of the presented work is determination of what constitutes a change in flexibility and what does not. That is, what degree of precision is present with the mDCM flexibility measures? This point is particularly important in this work because, using normal structure comparison metrics, the mutant dataset considered here is very similar to the wild-type structure. To address this point, we establish a baseline of ambient flexibility changes across a set of 7 wild-type structures [70], [71], [72], [73], [74], such that differences within the background profile arise from subtle differences in the wild-type X-ray structures (cf. **Table 3**). The baseline flexibility profile for each residue position for each residue FI value or pixel for CC is calculated as the average value over the set ±1 σ, where the standard deviation, σ, is respectively calculated over each data set at the corresponding residue or pixel. Then, any mutant flexibility metric within one standard deviation is considered “no change.” A value falling in the range between one and two standard deviations away from the mean defines “moderate” changes, whereas “large” changes are defined as greater than 2 standard deviations from the mean. As discussed above, **Fig. 1a** plots FI versus residue number for the wild-type baseline profile.

**Table 3:** Structural and thermodynamic characterization of the dataset.

Protein	PDBID	Resol. (Å)	R-value	RMSD (Å)	*T_m_* (K)	Max *C_p_*	Total # of HB	*u_sol_*	*v_nat_*
WT	1JWR	1.4	0.18	0.7	339	15.6	244	−2.13	−0.31
WT	1LZ1	1.4	0.18	0.6	339	17.5	240	−1.85	−0.14
WT	1LZR	1.5	0.14	0.5	339	15.5	250	−1.86	−0.21
WT	1LZS	1.6	0.17	0.7	339	16.3	244	−2.35	−0.37
WT	1REX	1.5	0.19	0.8	339	15.5	234	−2.00	−0.24
WT	1REY	1.7	0.17	0.8	339	15.1	229	−1.89	−0.12
WT	2NWD	1.0	0.13	–	339	15.5	238	−1.78	−0.19
**Average**		**1.4**	**0.17**	**0.68**	**339.0**	**15.9**	**239.8**	**−1.98**	**−0.23**
**Variation**		**15.4%**	**13.4%**	**17.1%**	**0.0%**	**5.1%**	**2.9%**	**10.1%**	**39.7%**
K1A	1C45	1.8	0.17	0.9	337	13.1	245	−1.66	−0.18
V2A	1OUG	1.8	0.17	0.8	333	16.8	229	−1.78	−0.26
Y38F	1WQO	1.8	0.17	0.8	338	18.8	229	−1.72	−0.20
Y45F	1WQP	1.8	0.17	0.8	337	18.5	231	−1.79	−0.28
Y54F	1WQQ	1.8	0.16	0.8	337	17.3	229	−1.86	−0.29
I56T	1OUA	1.8	0.15	0.8	325	14.8	243	−1.84	−0.28
Q58G	1B7R	1.8	0.16	0.7	345	19.0	235	−1.90	−0.30
I59S	2MEG	1.8	0.15	0.8	326	14.4	239	−1.96	−0.40
Y63F	1WQR	1.8	0.17	0.7	338	18.5	239	−1.86	−0.24
P71G	1LHI	1.8	0.16	0.8	336	20.3	240	−2.10	−0.33
V74A	1OUH	1.8	0.16	1.0	337	18.8	235	−1.76	−0.23
V100A	1OUB	1.8	0.16	0.7	337	18.2	232	−1.91	−0.36
P103G	1LHJ	1.8	0.15	0.8	339	18.2	231	−1.73	−0.18
Y124F	1WQM	1.8	0.16	0.8	338	19.0	230	−1.92	−0.32
**Average**		**1.8**	**0.16**	**0.80**	**335.9**	**17.6**	**234.8**	**−1.84**	**−0.28**
**Variation**		**0.0%**	**4.8%**	**9.8%**	**1.5%**	**11.8%**	**2.4%**	**6.2%**	**23.9%**

Note that all structures come from the same P 2_1_ 2_1_ 2_1_ space group. In the fifth column, the α-carbon RMSD of each structure is compared to the 2NWD wild-type structure after minimization, which is the structure closest to the centroid of the wild-type set. Maximum *C_p_* value in units of kcal/(mol·K). In all cases, δ*_nat_* is equal to 1.24.

The difference data presented in **Figs. 3** and **4** **–** **5** has been discretized into bins based on the above σ ranges. However, difference data in **Figs. 2** and **6** retain quantitative relative differences by setting the response in the change of flexibility to zero when it is within the noise level, and only allowing the signals to show up. In ΔFI_n_ and ΔCC_n_ the data is normalized in the following way:
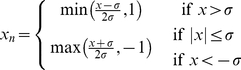
(5)The outcome of the above equation is that all values within the background profile are colored white, whereas continuous color schemes are used for the moderate change bins. The min() and max() functions are employed to threshold the coloring such that all “large” changes are colored the same maximum shade of red or blue. Further, because the values are normalized by context dependent standard deviations they in essence provide a degree of statistical significance for the observed change. That is, a change could be quantitatively large in raw values, but appear weak if the background variability was large. On the other hand, for extremely small standard deviations, the change will appear disproportionally large. However, this concern is largely unfounded as the per-pixel standard deviations in both ΔFI_n_ and ΔCC_n_ are relatively uniform (data not shown). In fact, plotting the raw differences actually makes changes appear roughly twice as frequent as we observe with the normalized scores, which would only strengthen the main conclusions of this paper. In other words, the normalized plots filter out response that does not have a signal large enough to distinguish against the background noise.

### Model Parameterization

The mDCM is parameterized by finding values of {*u_sol_*, *v_nat_*, δ*_nat_*} that best reproduce the experimental *C_p_* data using the same simulated annealing protocol previously employed [30]. Across the dataset, the resultant best-fit parameters are very similar. Nevertheless, we checked how the observed sensitivity is dependent on model parameterization. That is, a change in model parameters might change the nature of the FI and CC results, and potentially change the conclusions. To explore this concern, we first applied individual 3-parmater fits, and then fit the *C_p_* data using 2-free parameters per mutant while keeping the entropic parameter δ*_nat_* fixed across the dataset (cf. **Table 3**). Note that we used a similar strategy in prior works since the value of δ*_nat_* is related to protein fold [17], [18], [26], [29]. Encouragingly, the *C_p_* curves are again accurately reproduced (cf. **Fig. S1**), and the FI and CC values are both quantitatively consistent with the 3-parameter model. Furthermore, quantitatively similar FI and CC results are also obtained using a constant {*u_sol_*, *v_nat_*, δ*_nat_*} parameter set taken as the average over the 3-parameter best-fits (results not shown). For simplicity, the data presented throughout the report is solely based on the 2-parameter model, keeping in mind that the similar quantitative results arise from the other two-parameter sets.

The parameter differences observed in **Table 3** phenomenologically reflect physical differences between the mutants that are not explicitly considered by the model. For example, as we have demonstrated previously [26], parameter variation is expected to account for differences in hydrophobic interactions. The extent of parameter variation observed here is relatively small, generally within the variation expected for multiple equally good fits. Moreover, while thermodynamic quantities (i.e., *T_m_*) are somewhat sensitive to parameterization and input structure resolution, we have consistently demonstrated that mechanical FI and CC quantities are quite robust to parameter differences [26], [29], [30]. As such, the parameter differences have negligible affect on the presented results.

### Structure Preparation

In this work, we analyze X-ray crystal structures of 7 wild-type human c-type lysozymes and 14 spatially and chemically distinct point mutants. Each structure has been solved to high resolution (average = 1.8 Å), and all R-values are less than or equal to 0.19. PDBID's and all relevant structural information are provided in **Table 3**. There are ∼15 wild-type human lysozyme structures within the PDB. However, the series of cryogenic structures by Joti et al. [75] have extremely atypical properties, so we do not consider them here. In addition, the 1REZ [73] structure with a bound carbohydrate ligand also resulted in flexibility properties that were completely distinct from the remaining wild-type structures (and mutants for that matter). As such, it was also excluded, leaving the 7 considered structures. There are many more lysozyme point mutant structures present in the PDB than the 14 considered here; however, this dataset has been carefully selected so that the *C_p_* characterizations have been done under nearly identical experimental conditions [76], [77], [78], [79], [80], [81], [82]. Specifically, they have all been experimentally characterized using differential scanning calorimetry (DSC) under similar buffer conditions (pH = 2.7 to 2.8) and salt concentration (0.05 M). If this were not the case, model parameters would also reflect differences within the solvent conditions, thus obfuscating our direct comparisons. Moreover, full *C_p_* curves must also be available in the literature for us to fit to. Finally, the *C_p_* curves were generated by the same research group, which is important because DSC is a finicky technique that has systematic errors depending on differences in protocol and instrument. At the time of the writing of this paper, the 14 mutants studied here are the only ones that satisfy all of these criteria.

In all cases, hydrogen atoms are added using H++ server to ensure proper ionization [83] at the pH of the DSC experiments. The electrostatic parameters used are 0.05 M salinity and external/internal dielectrics of 80 and 6, respectively. Subsequently, the all-atom structures are minimized using the Molecular Operating Environment software using the Amber force field [84], which are then input into the mDCM.

## Supporting Information

Figure S1 Heat capacity best-fits for each structure using the employed 2-parameter (*u_sol_*, *v_nat_*) model. The native torsion entropy, δ*_nat_*, is determined by the average value from the 3-parameter best-fits, which is applied uniformly to all structures (cf. **Table 3**). Solid lines are model results, whereas points are experimental data.
(PDF)
