# Supplementary material for: Changes in Lysozyme Flexibility upon Mutation Are Frequent, Large and Long-Ranged
Source: PLoS Comput Biol. 2012 Mar 1;8(3):e1002409. doi: 10.1371/journal.pcbi.1002409 (PMC3291535; doi:10.1371/journal.pcbi.1002409)
Supplement: Figure S1 — Heat capacity best-fits for each structure using the employed 2-parameter (usol, vnat) model. The native torsion entropy, δnat, is determined by the average value from the 3-parameter best-fits, which is applied uniformly to all structures (cf. Table 3 ). Solid lines are model results, whereas points are experimental data. (PDF) [file pcbi.1002409.s001.pdf]

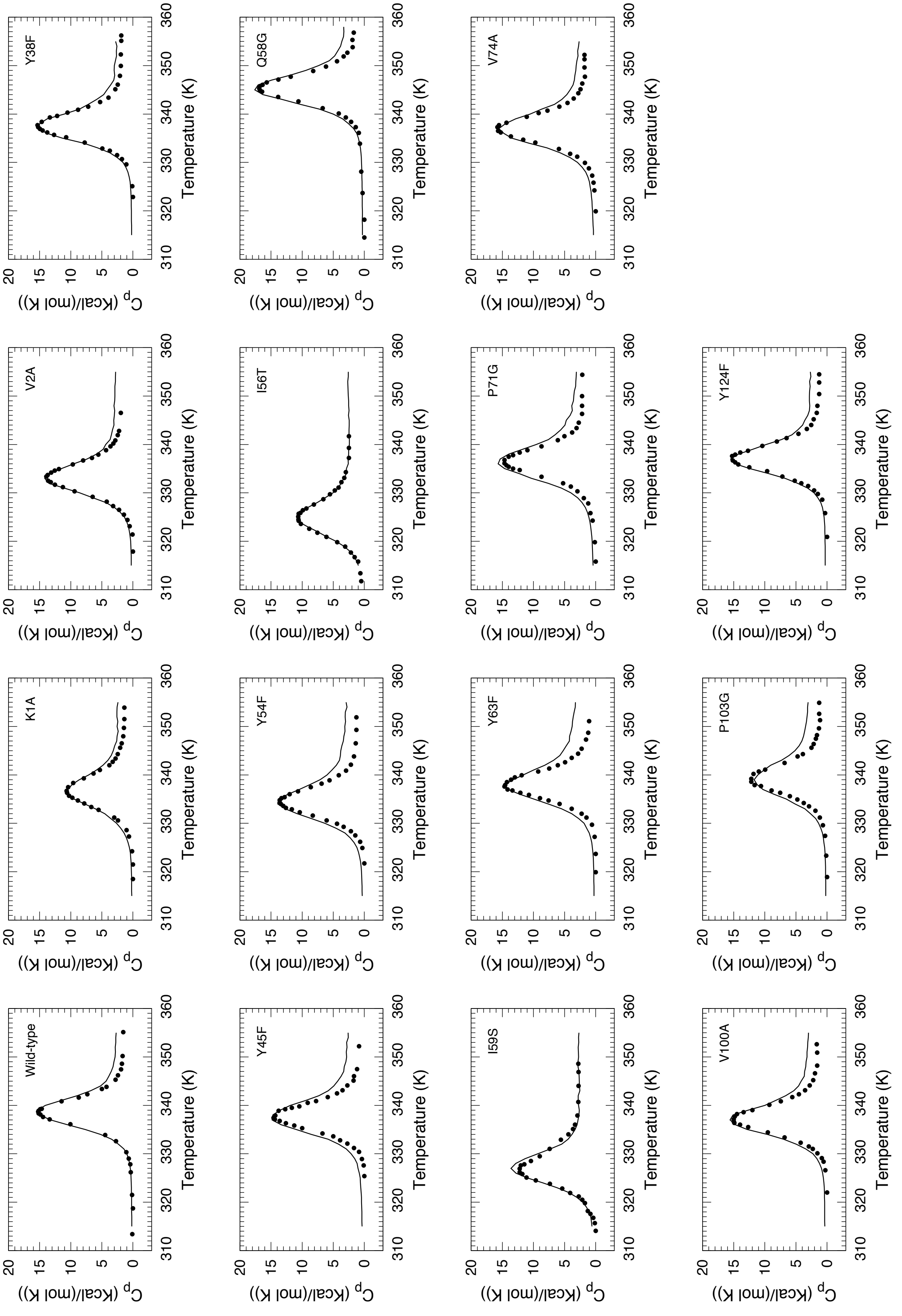

Figure S1. Heat capacity best-fits for each structure using the employed 2-parameter (u_{sol}, v_{nat}) model. The native torsion entropy, δ_{nat}, is determined by the average value from the 3-parameter best-fits, which is applied uniformly to all structures (cf. **Table 3**). Solid lines are model results, whereas points are experimental data.
